# Identification and characterization of screen use trajectories from late childhood to adolescence in a US-population based cohort study

**DOI:** 10.1016/j.pmedr.2023.102428

**Published:** 2023-09-21

**Authors:** Iris Yuefan Shao, Joanne Yang, Kyle T. Ganson, Fiona C. Baker, Jason M. Nagata

**Affiliations:** a Department of Pediatrics, University of California, San Francisco, San Francisco, CA, USA; b Factor-Inwentash Faculty of Social Work, University of Toronto, Toronto, Ontario, Canada; c Center for Health Sciences, SRI International, Menlo Park, CA, USA

**Keywords:** Digital media use, Adolescent health behavior, Growth mixture model, Perceived discrimination, Racial disparity

## Abstract

Screen use is a known risk factor for adverse physical and mental health outcomes during childhood and adolescence. Moreover, racial/ethnic disparity in screen use persists among adolescents. However, limited studies have characterized the population sharing similar longitudinal patterns of screen use from childhood to adolescence. This study will identify and characterize the subgroups of adolescents sharing similar trajectories of screen use from childhood to adolescence.

Study participants of the Adolescent Brain Cognitive Development Study (2016–2021) in the U.S with non-missing responses on self-reported screen use at each year of the study were included in the analysis. Growth mixture modeling was used to identify the optimal number of subgroups of adolescents with similar trajectories. Subsequently, socio-demographic characteristics, familial background, and perceived racism and discrimination during childhood was assessed for each subgroup population. Perceived discrimination was measured using the Perceived Discrimination Scale.

There were two major subgroups of individuals sharing similar trajectories of screen use: Drastically Increasing group (N = 1333); Gradually Increasing group (N = 10336). Higher proportions of the Drastically Increasing group were racial/ethnic minorities (70%) as compared to the Gradually Increasing group (45%). Moreover, the Drastically Increasing group had higher proportions of individuals reporting perceived racism and discrimination during childhood.

## Highlights

• There are two main subgroups of adolescents sharing similar screen use trajectory from late childhood to adolescence.
• Children that drastically increased screen use over time were more likely to be from disadvantaged backgrounds.
• Early adolescence is an important target time window for interventions that promote healthy screen use in adolescents.

## 1 Introduction

Changes in the social media landscape have drastically increased screen use among children and adolescents in the US (Vogels et al., 2022). However, existing studies on the effect of screen use on health and behavioral outcomes among children and adolescents yield mixed results (Browne et al., 2021, Maurer and Taylor, 2020). Evidence suggests that the impact of screen use on children varies by developmental stage, individual and familial characteristics (Browne et al., 2021, Maurer and Taylor, 2020). Moreover, studies have shown that screen use could be an important source of exposure to racism and discrimination in children, highlighting a need for better understanding of long-term screen use patterns among children from varying sociodemographic backgrounds and whether these behavioral patterns are associated with racism/discrimination (Tao and Fisher, 2022, Cohen et al., 2021). Therefore, this study aims to identify and characterize subgroups of adolescents sharing similar developmental trajectories of screen use from late childhood to adolescence using a national cohort of adolescents.

## 2 Methods

Participants of the Adolescent Brain Cognitive Development (ABCD) Study (2016–2021) with non-missing screen use data at any wave of the study follow-up were included in the analysis (N = 11,869). Average daily screen use was calculated as the sum of self-reported hours of daily use of television, videos, video games, social media, video chat, and texting (Bagot et al., 2018). Growth mixture modeling with standardized total screen use as the outcome and age as a continuous variable was used to identify the optimal number of subgroups of participants with similar trajectories. Subsequently, sociodemographic characteristics (baseline) and perceived racism and discrimination (year 1) was assessed for each subgroup population. All analyses were conducted in R (version 4.2.1) with ‘lcmm’ package (Proust-Lima et al., 2017). Centralized institutional review board approval was acquired from the University of California, San Diego. This study followed the Strengthening the Reporting of Observational Studies in Epidemiology reporting guideline. A parent/guardian and the child gave written informed consent and assent, respectively, to participate in the ABCD study.

## 3 Results

**Fig. 1:**
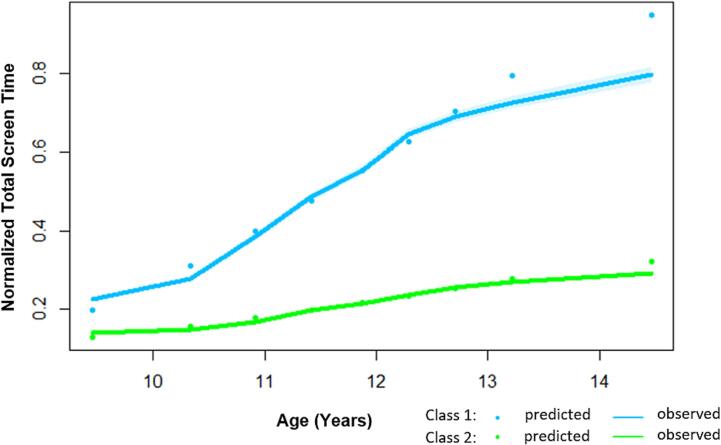
Trajectories of Screen Use from Late Childhood to Adolescence in the Adolescent Brain Cognitive Development Study.

49% of the eligible study population were female and 48% were racial/ethnic minorities. Based on maximum likelihood measures from the iterative model fitting and estimation (Supplemental Table 1), there were two distinct subgroups of individuals sharing similar trajectories of screen use from childhood to adolescence: Drastically Increasing group (N = 1,333) and Gradually Increasing group (N = 10,336) (Table 1**,** Fig. 1). The two groups had significantly different rates of increase in screen use between 10 and 11 years old and the difference slowly decreased starting in early adolescence around 13 years old. Higher proportions of the Drastically Increasing group were racial/ethnic minorities (70%) as compared to the Gradually Increasing group (45%). Moreover, the Drastically Increasing group had higher proportions of individuals reporting perceived racism and discrimination during late childhood.

**Table 1:** Class Member Descriptive Characteristics of Screen Use Trajectories from Late Childhood to Adolescence in the Adolescent Brain Cognitive Development (ABCD) Study, 2016–2021.

**N (%)**	**Class 1 (N = 1533) (Drastically Increasing)**	**Class 2 (N = 10336) (Gradually Increasing)**
**Female**	651 (43)	5025 (49)

**Race/ethnicity**		
Asian	47 (3)	662 (6)
Black	643 (42)	1751 (17)
Latino	289 (19)	1739 (17)
Native American	71 (5)	339 (3)
Other	25 (2)	128 (1)
White	457(30)	5714(55)

**Parental education**		
High School or Less	396 (26)	1645 (16)

Perceived racism/discrimination*		
Perceived been treated unfairly or negatively because of ethnic background by:		
Teachers	104 (8)	259 (3)
Adults outside school	81 (6)	251 (3)
Peers	272 (20)	941 (10)
“I feel I am not wanted in American Society”	56 (4)	158 (2)
“I don’t feel accepted by other Americans”	57 (4)	211 (2)
“I feel that other Americans have something against me”	83 (6)	274 (3)
“Others behave in an unfair or negative way toward my ethnic group”	174 (13)	598 (7)
Discrimination because of race, ethnicity, or color	119 (9)	353 (4)

*: Measures for perceived racism/discrimination were from the Perceived Discrimination Scale.

## 4 Discussion

In this large, diverse national sample of adolescents, we identified two subgroups of adolescents sharing similar developmental trajectories of screen use from late childhood to adolescence. Our study showed that children that drastically increased screen use over time were more likely to be racial/ethnic minor and coming from households with lower parental education level. This finding is consistent with existing study that reported an association between higher screen use and disadvantaged background (Männikkö et al., 2020). Consistent with a previous study (Bitto Urbanova et al., 2020), our study showed that higher proportions of children that drastically increased their screen use during early adolescence reported perceived racism and discrimination during childhood. Moreover, our study highlighted that early adolescence between 11 and 13 years old is an important target time window for behavioral interventions that promote healthy screen use in adolescents. Future studies should assess key factors that shape the trajectories of screen use across developmental periods further into later adolescence to inform guidance for pediatricians, parents, and adolescents.

## 5 Funding/Support

Jason M. Nagata was funded by the National Institutes of Health (K08HL159350), the American Heart Association Career Development Award (CDA34760281), and the Doris Duke Charitable Foundation (2022056).

## 6 Additional information

The ABCD Study was supported by the National Institutes of Health and additional federal partners under award numbers U01DA041022, U01DA041025, U01DA041028, U01DA041048, U01DA041089, U01DA041093, U01DA041106, U01DA041117, U01DA041120, U01DA041134, U01DA041148, U01DA041156, U01DA041174, U24DA041123, and U24DA041147. A full list of supporters is available at https://abcdstudy.org/federal-partners/. A listing of participating sites and a complete listing of the study investigators can be found at https://abcdstudy.org/principal-investigators.html. ABCD consortium investigators designed and implemented the study and/or provided data but did not necessarily participate in the analysis or writing of this report.

## 7 Role of funder/sponsor (if any)

The funders had no role in the design and conduct of the study; collection, management, analysis, and interpretation of the data; preparation, review, or approval of the manuscript; and decision to submit the manuscript for publication.

## 8 Clinical trial registration (if any)

Not Applicable.

## Declaration of Competing Interest

The authors declare that they have no known competing financial interests or personal relationships that could have appeared to influence the work reported in this paper.

## Data Availability

Data will be made available on request.
