# Supplementary material for: Identification and characterization of screen use trajectories from late childhood to adolescence in a US-population based cohort study
Source: Prev Med Rep. 2023 Sep 21;36:102428. doi: 10.1016/j.pmedr.2023.102428 (PMC10520867; doi:10.1016/j.pmedr.2023.102428)
Supplement: Supplementary data 1 [file mmc1.docx]

**Supplemental Table 1. Model Selection Criteria for Screen Use Trajectories from Late Childhood to Adolescence in the Adolescent Brain Cognitive Development Study**

| **Screen Time Score** |  | | |
| --- | --- | --- | --- |
|  | **AIC** | **BIC** | **Entropy** |
| **1-class model** | -44892.38 | -44818.56 | 1.00 |
| **2-class model *** | -47085.77 | -46982.42 | 0.79 |
| **3-class model** | -47077.77 | -46944.89 | 0.36 |
| **4-class model** |  |  |  |
|  |  |  |  |
| **Optimal Model Class Membership** |  | | |
|  | **Class 1** | **Class 2** | |
| **N (%)** | 1533 (13) | 10336 (87) | |
| **Distribution of posterior probability of class membership** | **1st Quartile** | **Mean** | |
| Class 1 | 0.74 | 0.85 | |
| Class 2 | 0.97 | 0.96 | |
| *: 2-class model is final model. |  |  | |
